# Virus-like particle vaccinology, from bench to bedside

**DOI:** 10.1038/s41423-022-00897-8

**Published:** 2022-08-12

**Authors:** Mona O. Mohsen, Martin F. Bachmann

**Affiliations:** 1 Department of BioMedical Research, University of Bern, Bern, Switzerland; 2 Department of Immunology RIA, University Hospital Bern, Bern, Switzerland; 3 Saiba Biotech AG, Bahnhofstr. 13, CH-8808 Pfaeffikon, Switzerland; 4 The Jenner Institute, University of Oxford, Oxford, UK

**Keywords:** vaccine, virus-like particle, immunology, Vaccines, Immunology

## Abstract

Virus-like particles (VLPs) have become key tools in biology, medicine and even engineering. After their initial use to resolve viral structures at the atomic level, VLPs were rapidly harnessed to develop antiviral vaccines followed by their use as display platforms to generate any kind of vaccine. Most recently, VLPs have been employed as nanomachines to deliver pharmaceutically active products to specific sites and into specific cells in the body. Here, we focus on the use of VLPs for the development of vaccines with broad fields of indications ranging from classical vaccines against viruses to therapeutic vaccines against chronic inflammation, pain, allergy and cancer. In this review, we take a walk through time, starting with the latest developments in experimental preclinical VLP-based vaccines and ending with marketed vaccines, which earn billions of dollars every year, paving the way for the next wave of prophylactic and therapeutic vaccines already visible on the horizon.

## Introduction: the concept of virus-like particles (VLPs)

The term virus-like particles (VLPs) refers to particles that self-assemble as a result of the expression of proteins encoding capsids, cores or envelops of viruses or even preparations of monolayered particles derived from a multilayered virus [1]. Symmetrical particles formed from nonviral or artificial proteins can also be considered VLPs [2, 3]; in this case, symmetry refers to the way the capsomere units are geometrically organized. However, this category of VLPs is not discussed in this review. Additionally, VLPs self-assemble into particles that resemble or mimic the structure, size, and symmetry of original viruses, however VLPs cannot replicate as they lack a genome and replicases [1]. Detailed descriptions of VLP structure, immunogenicity and expression as they relate to vaccination are reviewed elsewhere [4, 5].

The structural proteins of hepatitis B virus, mainly the core (HBc) and the surface antigen (HBsAg), were among the first VLPs to be expressed in heterologous expression systems. These efforts resulted in the first recombinant human vaccine in 1986 against HBV that utilizes surface antigens [6]. Next, a vaccine against human papillomavirus (HPV), which causes cervical cancer, that uses the L1 structural protein was introduced. HPV vaccines entered the market in 2006 and 2007 [7, 8]. Following this success, a vaccine against hepatitis E virus (HEV) was approved in 2011 in China [9]. Overall, the use of VLPs as a conventional vaccine platform possesses several advantages, as listed in Table 1.

**Table 1:** Advantages of using conventional VLPs as a vaccine platform

Advantage	Explanation
Safety	VLPs lack the ability to replicate due to the absence of replicases and nucleic acids that encode viral proteins [159].
Symmetry	Usually, the symmetry of VLPs reflects the symmetry of the parental or original virus [173].
Flexibility in assembly	Typically, the capsid/envelope proteins assemble into VLPs, but core proteins may also form VLPs. A famous example is HBV as surface proteins assemble into HBsAg-VLPs and core proteins assemble into HBcAg-VLPs [173].
Assembly/disassembly process	Some VLPs can spontaneously assemble into icosahedral particles around nucleic acids. For instance, the bacteriophage Qβ naturally assembles into icosahedral particles of ~30 nm upon expression in *Escherichia coli* (*E. coli*). Qβ-VLPs can be disassembled and will spontaneously reassemble in the presence of polyanionic structures [159].
Packaging the interior surface of VLPs	ssRNA packaged into VLPs upon expression in *E*. *coli* is a potent TLR7/8 ligand. VLPs can be reassembled in the presence of oligodeoxynucleotides such as CpGs, a TLR9 ligand, dsRNA, a TLR3 ligand or polyGlu which does not bind to any TLR [173].
Decorating the exterior surface of VLPs	The surface of VLPs can be efficiently decorated with target epitopes using different chemical and genetic fusion techniques [174]. The repetitive surface features allows high densities of the target antigen to be displayed [175].
Surface structure	The repetitive surface geometrical structure of VLPs is considered a pathogen-associated structural pattern (PASP) which is a potent inducer of antibody response [14].
The role of size	Particulate antigens 20–200 nm in size such as VLPs rapidly drain to lymph nodes (LNs) and interact with antigen-presenting cells (APCs) and B cells [5, 130]. This feature mediates effective stimulation of B and T-cell responses.
Expression systems	Various expression systems can be utilized to generate VLPs, including bacteria, yeast, mammalian cells, insect cells and plants.
Stability	Generally, VLPs are stable; nevertheless, improving their stability would improve vaccine deployment logistics. Methods to enhance stability and extend shelf-life include the introduction of intersubunit disulfide bonds [118, 176] and lyophilization or spray-drying during postproduction phase [119, 120].
Large-scale production	Cost-effective and robust large-scale production is feasible for many VLPs [32, 121].
Examples of commonly used VLPs in vaccine development	HBV, HPV, Qβ, CuMV, AP205, CCMV, MS2, PP7, RHDS, and CPV

*CuMV* cucumber mosaic virus, *HBV* hepatitis B virus, *CCMV* cowpea chlorotic mosaic virus, *RHDS* rabbit hemorrhagic disease virus, *CPV* canine parvovirus.

The current review aims to discuss some recent updates in VLP-based vaccine development by first describing some examples of VLP-based vaccines in preclinical development and clinical trials and finally describing the VLP-based vaccines currently on the market.

## Preclinical development

VLPs have been extensively used as prophylactic or therapeutic vaccine platforms for a wide range of diseases. In this section, we aim to discuss some examples of the preclinical application of VLPs as a vaccine platform for successful induction of antibodies and/or T-cell responses in both prophylactic and therapeutic regimens in the following diseases: infectious diseases (examples discussed: Middle East respiratory syndrome, coronavirus disease 2019 (COVID-19), influenza, malaria and acquired immunodeficiency disease (AIDS), inflammation, allergy, pain, neurodegenerative diseases (Alzheimer’s and Parkinson’s disease) and cancer).

### VLP-based vaccines for the induction of antibodies in prophylactic or therapeutic regimens

**Fig. 1:**
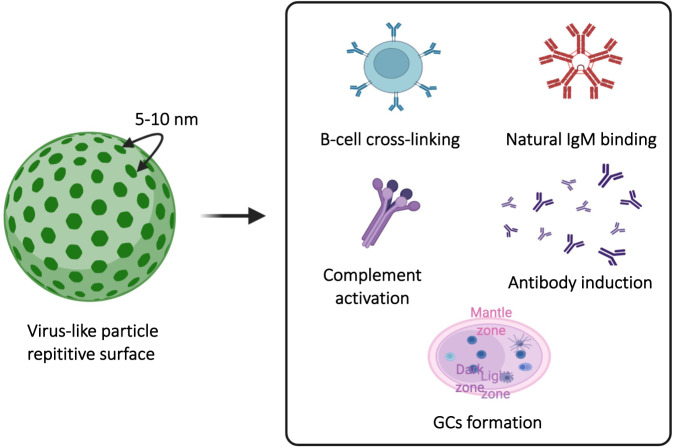
Antigen processing is facilitated when antigens are particulate with repetitive surface epitopes spaced every 5–10 nm. Such repetitive structures are recognized by the immune system as Pathogen associated structural paterns (PASPs), which facilitate the cross-linking of B cells, natural IgM binding, complement activation, high-affinity long-lived antibody induction and GC formation. Created with BioRender.com

B cells can be optimally stimulated by particles with repetitive surfaces. It was found in the 1970s that an optimal B-cell response can be induced by at least 12–16 epitopes spaced 5–10 nm apart (e.g., haptens and artificial polymers), referred to as immunons [10, 11]. It was subsequently realized that typical RNA viruses exhibit the characteristics of multiple immunons, as they often have a diameter of 30 nm and 180 copies of a coat protein spaced ~5 nm apart, as illustrated in Fig. 1 [10]. The advantages of using such particles include the efficient cross-linking of B-cell receptors [10] and successful recognition by natural IgM antibodies that results in the activation of the classical complement pathway, causing the deposition of antigens on follicular dendritic cells (FDCs) [5, 12], enhancing germinal center (GC) formation [13], and inducing durable and long-lived plasma cells [14]. Accordingly, VLPs decorated with full-length antigens and loaded with RNA are considered an important tool for activation of B-cells and induction of long-lived B-cell responses [11]. In addition, for some VLPs, in particular RNA virus-derived VLPs, loading the particle with RNA or CpGs (DNA oligonucleotides rich in nonmethylated CG motifs) is possible, which results in TLR7/8 or TLR9 activation, respectively, in B cells. This augments IgG2a and IgA antibody responses and enhances plasma cell formation [15] and affinity maturation [16]. The type of RNA packaged in VLPs also matters, with bacterial RNA being the most efficient B-cell enhancer [17]. Below, we discuss some examples of prophylactic and therapeutic VLP-based vaccines that elicit efficient antibody responses, thus mediating protection against disease. In cases where the targeted antigen does not induce good T_H_ cell responses (e.g., self-molecules or carbohydrates), T_H_ against the VLPs is essential, as it will mediate intermolecular interactions, driving antibody responses against the targeted antigen as well.

#### Infectious disease

##### Coronavirus disease

**Fig. 2:**
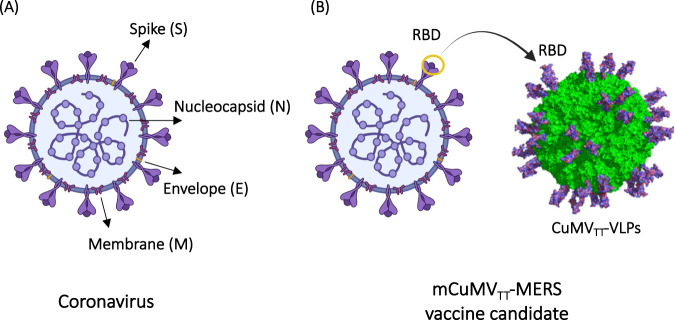
**A** A typical coronavirus based on four structural proteins: spike (S), envelope (E), membrane (M) and nucleocapsid (N). **B** Strategy of developing a mosaic VLP-based vaccine by genetically fusing the RBD of MERS-CoV into the optimized CuMV_TT_-VLPs, which incorporate a universal TT epitope and TLR7/8 ligand. Created with BioRender.com

Coronaviruses are a family of viruses that have recently attracted much attention, mainly due to the ongoing pandemic caused by SARS-CoV-2. Coronaviruses are RNA viruses characterized by a single-stranded positive RNA molecule of approximately 30 kB, encoding, among others, four structural proteins, as illustrated in Fig. 2A: spike (S), membrane (M), nucleocapsid (N) and envelope (E). Extensive efforts have been made to develop vaccines against this family of viruses using conventional VLP platforms. However, the majority of these efforts are still in the preclinical stage, with the exception of some candidates against SARS-CoV-2 that are currently in phases I, II and III, as summarized in Table 2.

*Middle East Respiratory Syndrome (MERS):* The Middle East respiratory syndrome coronavirus (MERS-CoV) causes severe respiratory disease in humans and continues to be a threat in more than 27 countries worldwide following the first registered pandemic in Saudi Arabia ten years ago. The mortality rate of MERS is very high and in the range of 40% [18]. MERS-CoV contains several immunogenic proteins, including the spike (S) protein, which mediates virus entry into host cells via its receptor dipeptidyl peptidase 4 (Dpp4). Accordingly, the receptor is used as the principle targeting agent against the virus in vaccine development. Preclinical studies were performed to develop vaccines against MERS-CoV using different platforms, including VLPs [19, 20].

We have recently optimized a plant-derived VLP called cucumber-mosaic virus-like particles (CuMV_TT_) as an improved vaccine platform incorporating a tetanus toxin (TT) epitope by genetic fusion. Incorporation of the TT epitope is thought to enhance the interaction between B cells and T_H_ cells, especially in elderly people, due to the presence of preexisting TT-specific memory T_H_ cells. Furthermore, CuMV_TT_-VLPs incorporate RNA from *E*. *coli*, which serves as a TLR7/8 agonist, during the expression process. This platform has proven to be highly immunogenic in mice, cats, dogs, rabbits and horses [21–24].

Using this new platform (CuMV_TT_), we have developed a scalable vaccine based on genetic fusion of the receptor-binding motif (RBM) of the MERS-CoV spike protein into CuMV_TT_-VLPs (Fig. 2B). The resultant mosaic VLP-based vaccine (mCuMV_TT_-MERS) was expressed in *E. coli*, allowing spontaneous packaging of the TLR7/8 ligand. mCuMV_TT_-MERS induced high levels of specific antibodies capable of neutralizing MERS-CoV/EMC/2012 isolate, demonstrating promising potential for translation into dromedaries and humans [25]. Wang et al. constructed a MERS-CoV-VLP vaccine using a baculovirus expression system that was tested in rhesus macaques. The developed vaccine could induce specific anti-RBD titers (ELISA) of 1:1280 in addition to T-cell-mediated immunity. The study demonstrated that MERS-CoV-VLPs have excellent immunogenicity and are promising vaccine candidates [26]. The same group also developed a chimeric VLP-based vaccine expressing RBD of MERS-CoV utilizing VP2 structural protein of canine parvovirus (CPV). Immunization with the chimeric vaccine also induced RBD-specific humoral and cellular immunity in murine models [27]. Unfortunately, no VLP-based vaccine has progressed to clinical trials thus far.

*Severe Acute Respiratory Syndrome (COVID-19):* The ongoing COVID-19 pandemic has overwhelmed the world’s health systems. Approximately thirty different vaccines have been approved for human use in different countries, and approximately eighty candidates are in clinical development, of which 19 are in phase III trials [28]. Nevertheless, with the continuous emergence of new variants of concern (VOCs), there is still a need for the development of effective, scalable, affordable and stable classical vaccines.

**Fig. 3:**
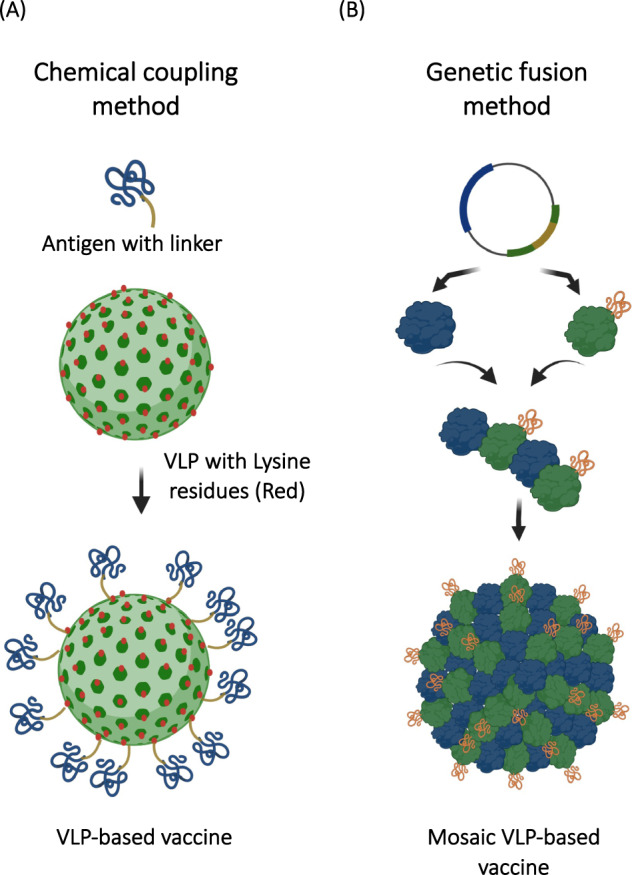
Schematic presentation of the following techniques. **A** Chemical techniques to conjugate an epitope to VLPs are mainly based on modifying a chemical group on the surface of the VLP. Lysine (Lys) residues on the surface of a VLP (shown as red dots) are the most commonly used target for modification. Epitopes should be modified to contain a free Cys for the chosen chemistry conjugation method. **B** A genetic fusion technique allowing the coexpression of both the VLP capsid proteins and the targeted epitope. Genetic fusion techniques may be considered a better option for GMP translational purposes and scaling up [37]. Created with BioRender.com

As an example, we have developed a COVID-19 vaccine by chemically coupling the receptor-binding domain (RBD) of SARS-CoV-2 to our immunologically optimized CuMV_TT_ VLPs. We have utilized the most popular target for modification on the VLP surface, which contains accessible lysine residues (Fig. 3A). The displayed RBD recognized the viral receptor angiotensin-converting enzyme 2 (ACE2), confirming the correct conformational structure and resulting in a highly specific antibody response that neutralized SARS-CoV-2 [29] and VOCs [30] in vitro. A similar platform, CuMV_TT_-RBD, was tested for intranasal administration in a murine model. The vaccine candidate elicited a strong specific systemic and mucosal IgG and IgA response that efficiently neutralized different VOCs [31].

For translational purposes, we next designed a novel vaccine candidate by genetically grafting RBM of SARS-CoV-2 into CuMV_TT_-VLPs, called mosaic CuMV_TT_-RBM (Fig. 3B). The developed vaccine could be produced on a large scale (>2.5 million doses per 1000-liter fermenter run), showed high stability at 4 °C for 14 months and may be used for multiple booster doses. CuMV_TT_-RBM also induced high specific anti-RBD and anti-spike antibody titers in murine and rabbit models that were cross-reactive to mutant RBDs and VoCs [32]. Due to the high production rate and ease of storage at 4 °C, we expect that production and shipment costs will be orders of magnitude lower than for current RNA-based vaccines. Similar results were obtained with RBM fused to VLP AP205 [33]. In addition, we generated a double mosaic particle, which induced neutralizing antibodies against both RBM in S1 as well as the cleavage site in S2, a second, “minor” neutralizing epitope [34].

Using different techniques, other groups have also successfully developed vaccines against COVID-19 utilizing VLPs. For instance, Tan et al. designed and tested RBD-SpyAP205 vaccine in a prime/boost regimen in murine models. They used SpyTag/SpyCatcher technology to display RBD on the AP205-VLP platform. Their results showed that the vaccine candidate can elicit a neutralizing antibody response [35].

As discussed above, SARS-CoV-2 contains four essential structural proteins that can assemble into VLPs in mammalian cells without the viral genome. Yilmaz et al. described a VLP-based vaccine expressing hexaproline prefusion-stabilized spike (S-6p) as well as N, M and E structural proteins of SARS-CoV-2. The vaccine was absorbed to alum and formulated with K-type CpG ODN [36]. HEK293 cells were used to express the vaccine candidate, and vesicular VLPs that expressed the four structural proteins were generated. The results demonstrated effective production of anti-S, anti-RBD and anti-N IgG and prevention of lung pathology in vaccinated animals. This vaccine candidate is currently in a phase II clinical trial; please refer to Table 2.

**Fig. 4:**
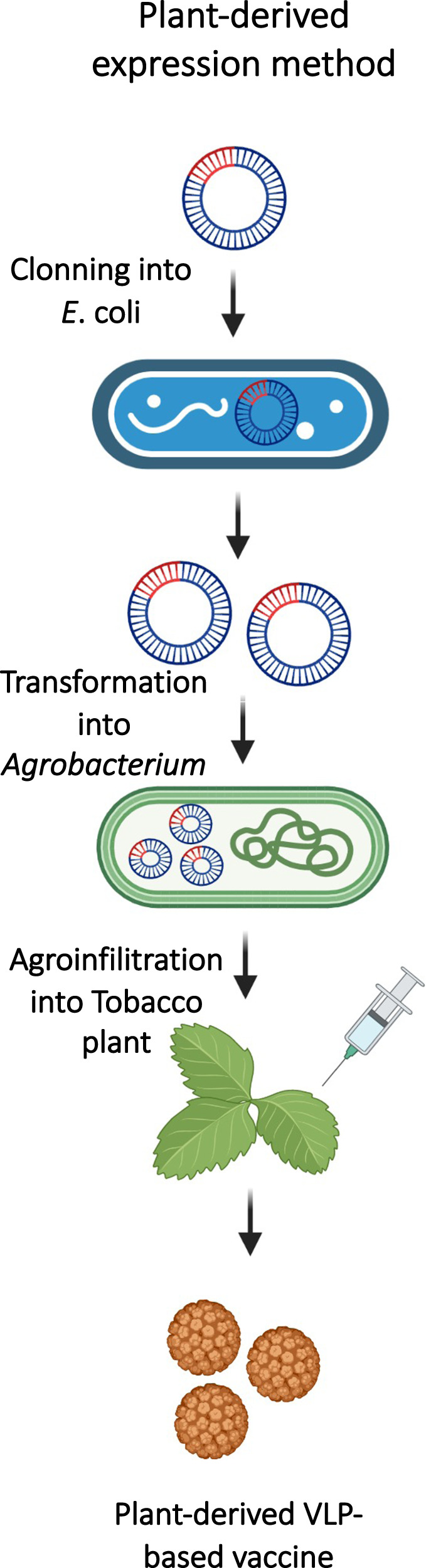
Agroinfiltration method for co-expression process using two vectors in tobacco plants, adapted from (37). Created with BioRender.com

In general, recombinant protein-based vaccines can be produced using different host expression systems, such as insect cells, yeast, mammalian cells and plants. Recently, plant-based vaccine development has become more accepted in the field following the COVID-19 pandemic. A recently published study described the successful construction of a SARS-CoV-2 vaccine based on VLPs in plants. The authors cloned the M, N and E genes of SARS-CoV-2 into a geminiviral vector and transiently expressed them in *N. benthamiana* plants. The process resulted in plant-derived VLPs that exhibited a similar shape and size as the native SARS-CoV-2 virion, without the spike protein. Figure 4 illustrates a plant-expression system process using the agroinfilitration method in tobacco plants. The authors indicated that such VLPs could serve in the future as a platform to carry S antigens as well [37].

The plant-derived VLP-production method has also been utilized by Medicago Inc. for the development of their SARS-CoV-2 vaccine (CoVLPs vaccine; Covifenz). CoVLPs were produced from the expression of a modified full-length S protein, which, upon expression in plant cells, trimerizes and moves to lipid rafts in the plasma membrane to spontaneously assemble into VLPs that “bud” off the surface of the plant cell. In macaques, these VLPs induced strong and lasting IgG responses, particularly in the presence of AS03 adjuvants, and boosted T-cell responses, resulting in protection of primates against viral infection [38]. CoVLPs are currently in late-stage clinical trials and have been approved in Canada for use in humans (Table 2).

##### Influenza

**Fig. 5:**
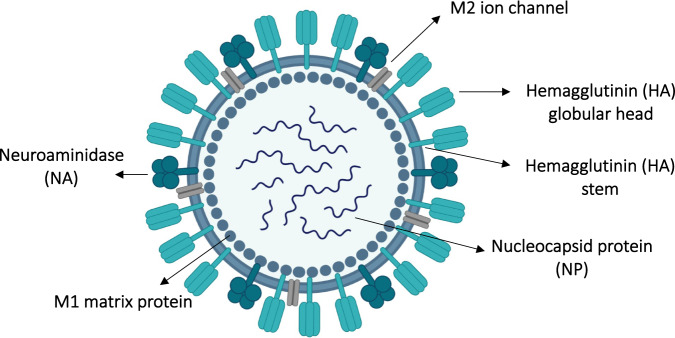
A cartoon illustrating the major antigens of Influenza virus including: hemagglutinin (HA) globular head and stem, M1 matrix protein, M2 ion channel, NA neuraminidase and nucleocapsid protein (NP). Created with BioRender.Com

Influenza virus is an enveloped RNA virus belonging to the family Orthomyxoviridae. The virus surface glycoproteins hemagglutinin (HA) and neuraminidase (NA) are the major targets for vaccine development. HA consists of a globular head and a stem. The current marketed seasonal vaccines against influenza target the globular head of HA for neutralization; however, rapid antigenic variation in the globular head abrogates the affinity of the induced antibodies, making yearly or biyearly development of novel vaccines necessary [39]. Conversely, the stem of HA is more conserved; however, due to the immunodominance of the globular head and the steric hinderance of the stem, it may be challenging but not impossible to mount an efficient immune response against the stem [40]. Targeting NA, the second major protein in influenza virus, can prevent virus entry into the host cell, decrease disease severity, and inhibit virus replication [39]. Similar to HA and NA, the M2 protein is an integral surface protein of influenza virus, and the extracellular domain of M2 is highly conserved in influenza A [41]. Matrix protein (M) and nucleoprotein (NP) are the major internal proteins of the virus. Fig. 5 illustrates the structure and major antigens of influenza virus.

VLPs constitute an attractive, alternative vaccine platform to traditional influenza vaccine formulations, as they mimic the native virus but lack any genetic materials. Buffin et al. showed that the use of mammalian cells can offer many advantages for vaccine production, including the maintenance of glycosylation patterns. They developed a VLP vaccine containing HA, matrix (M1) and NA proteins. The expressed VLPs emulate the exterior surface of the authentic virus and showed high immunogenicity [42].

Using the bacteriophage Qß, we designed a VLP vaccine against various forms of the globular domain of HA. The globular domains were chemically coupled to the surface of the VLP [43]. Essentially, all vaccine candidates induced protective antibodies in mice that also cross-reacted with drifted strains. Interestingly, the HA globular domain was produced in bacteria, indicating that glycosylation was not essential for the induction of protective antibodies, as levels of hemagglutination inhibiting antibodies in mice were similar to those obtained with a classical vaccine [44]. In a next step, this vaccine was tested in a phase I study, revealing that this fully bacterial-produced vaccine was at least as immunogenic in humans as a classical, virus-based vaccine for both antibodies [45] and T cells [46]. Another interesting approach is based on HA displayed on ferritin nanoparticles, which induce high levels of broadly neutralizing antibodies against H1N1 influenza virus strains [47].

As HA proteins are very variable and poorly conserved, a vaccine based on conserved structures such as the extracellular domain of M2 (eM2) or the HA stem may be an interesting alternative to HA- and NA-based vaccines. Indeed, early experiments displaying eM2 on HBcAg by genetic fusion [41, 48, 49] or Qβ by chemical conjugation [50] induced antibodies that could protect against multiple clades of influenza A viruses. The Qβ-based M2e vaccine was also tested for its ability to induce protective antibodies upon intranasal administration. Vaccination using this route induced high levels of protective IgG and IgA antibodies in the serum and lungs of mice [51].

Similar to Qβ, AP205-VLPs derived from the bacteriophage AP205 have also shown high induction of specific antibodies against M2 protein displayed at the N-terminus by genetic fusion [52]. In the context of eM2-based vaccines against influenza virus, it is interesting to note that we have provided evidence in a murine influenza model that VLPs carrying prokaryotic RNA, a TLR7/8 ligand, and displaying eM2 induced better protective IgG subclasses than VLPs carrying eukaryotic RNA [17], highlighting the importance of the type of packaged RNA and confirming that the subclass of anti-M2 IgG antibodies is essential for protection [53].

The stem region of HA, which is more conserved than the globular domain of HA, may be an additional attractive target. Indeed, it is possible to express the stem region as a trimer suitable for chemical conjugation to VLPs [54], and when displayed on HBcAg, it can induce strong IgG responses in mice [55]. In addition, HA stem displayed on ferritin nanoparticles induced broadly neutralizing and protective antibodies in mice [56]. Thus, VLP-based vaccine candidates targeting conserved structures of influenza virus, HA or M2 proteins, may be a suitable alternative to the currently used influenza vaccines for battling seasonal and future influenza pandemics [57].

As an alternative method to induce broader immune coverage against influenza subtypes, a mosaic, multisubtype VLP vaccine has been developed containing 3 or 4 full-length HA proteins from H5N1, H7N2 and H9N2. The multiclade VLP vaccines were tested intranasally in ferrets and showed efficacy and immunogenicity [58]. Several VLP-based vaccines against H9N2 have also been described [59].

##### Malaria

The WHO estimated that there were 241 million malaria cases in 2020 in approximately 85 malaria-endemic countries in comparison to 227 million cases in 2019 [60]. Mortality cases increased in Sudan by 49% between 2016 and 2020, and 80% of reported cases were due to *Plasmodium falciparum* (*P. falciparum)* [60]. Vaccination against malaria can be categorized into three approaches: preerythrocytic vaccines, blood-stage vaccines, and transmission-blocking vaccines (TBV) [61]. The most advanced preerythrocytic vaccine is RTS,S (Mosquirix^TM^), a VLP-based vaccine that was recommended by the WHO in October 2021 for use in high-risk areas [60]. RTS,S is discussed below in Section “Marketed VLP-based vaccines”.

Chan et al. designed and developed a transmission-blocking vaccine by utilizing duck HBV VLPs that incorporate large Pfs230 and Pfs25 proteins. The vaccine was expressed in the Hansenulla cell line compatible with cGMP. Induced antibodies could recognize the native protein on the gametocyte surface and reduce the transmission activity in a standard membrane feeding assay [62]. The same group recently used the small surface antigen (dS) of duck HBV to display circumsporozoite protein (CSP) in a larger portion on the surface of the scaffold. With the goal of overcoming RTS,S drawbacks, CSP is present on only a small portion of the vaccine surface [63]. The Barillas Mury group has also focused on developing a TBV by conjugating Pfs47 (a protein expressed on the surface of gametocytes) to an Acinetobacter phage (AP205-VLPs) using the Spycatcher-SpyTag technique. The candidate vaccine induced a high antibody titer with high affinity for Pfs47, confirming its potential [64].

#### Inflammation

VLPs have been used extensively for the development of therapeutic vaccines against different chronic inflammatory diseases. In this respect, proofs of concept for different preclinical vaccines in different animal species have been generated.

**Fig. 6:**
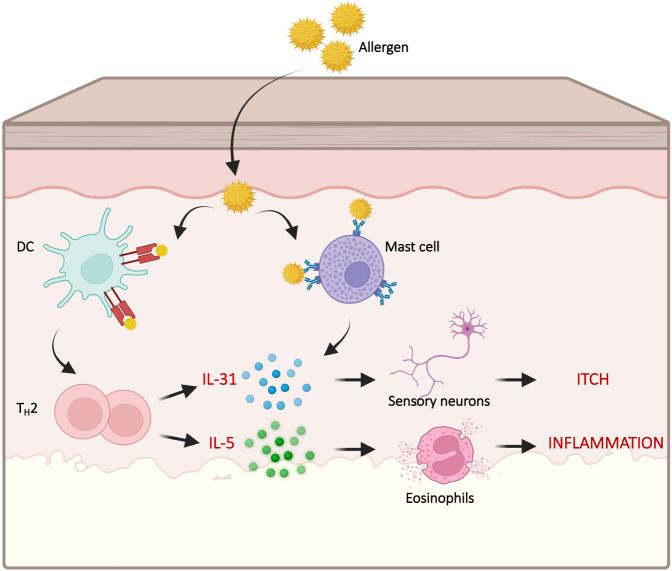
IL-31 and IL-5 are central mediators of allergic itching. Typically, allergens penetrate the skin triggering mast cells and T-helper cells type 2 (TH2). Secretion of IL-31 stimulates peripheral sensory neurons which results in itching. IL-5 is the master cytokine required for both generation and activation of eosinophils. Created with BioRender.com

In insect-bite hypersensitivity (IBH), horses suffer from chronic allergic dermatitis caused by type-I/type-IV allergic reactions mediated by eosinophils and caused by midge (Culicoides) bites. Interleukin-5 (IL-5) is considered a key stimulator of eosinophils [65, 66] (Fig. 6).

Monoclonal antibodies (mAbs) against IL-5 are successfully used to treat human diseases with an eosinophilic component, particularly eosinophilic asthma [67]. It may therefore be expected that lowering levels of IL-5 by vaccination reduces eosinophilic diseases in general, including eosinophilic infiltration of the horse skin in IBH [68], extending earlier observations in mice [69, 70]. To this end, we covalently linked equine IL-5 to our CuMV_TT_ platform and tested the vaccine in a placebo-controlled study of thirty-four Iceland horses. The vaccine was formulated without adjuvants and did not show any safety concerns but induced anti-IL-5 autoantibodies in 89% of the vaccinated horses, which translated into clinical improvement [71]. Efficacy rates could be increased to 100% and maintained over several seasons by yearly booster injections, essentially eliminating symptoms almost completely [68]. This approach may be considered a breakthrough therapy targeting a chronic inflammatory disease in horses and has the potential for future translation to humans.

In addition to IL-5, IL-31 likewise plays a major role in IBH as well as in allergic pruritis in humans, dogs, monkeys and mice (Fig. 6). In contrast to IL-5, IL-31 mainly promotes itching; this itching, however, this results in chronic scratching causing local inflammation, typically exacerbated by infection [72]. Based on CuMV_TT_ platform, an IL-31 equine vaccine was generated and tested in horses. The results demonstrated the safety of the vaccine and revealed the efficacy of the approach, resulting in strongly reduced clinical scores in the treatment group compared with the placebo group [24]. Atopic dermatitis is the most common allergic disease in dogs, causing extensive scratching and loss of fur as well as secondary infections. Canine IL-31 displayed on CuMV_TT_ was tested in house dust mite-sensitized dogs who developed typical symptoms of atopic dermatitis upon topical challenge with allergen. Immunized dogs showed strongly reduced itching following immunization, which correlated with the induced specific antibody titers. However, a less pronounced response was noticed upon the decline in antibody titers, indicating that maintenance of IgG titers is an area for future improvement [23]. The performed study lays the foundation for a therapeutic modality for the effective treatment of atopic dermatitis in dogs. Further studies to increase and prolong the efficacy have yet to be performed, and the results with monoclonal anti-IL-31 antibodies will direct the path of the development of a vaccine targeting IL-31 in humans.

In addition to IL-5, VLPs displaying IL-13 may be an additional interesting modality to be broadly used to treat atopic dermatitis and are an avenue that should be explored in more detail together with VLPs targeting IL-4 [73].

It has been shown that mAbs generated against the proinflammatory cytokine IL-17A are highly effective against psoriasis, which affects between 1 and 4% of global populations [74]. The high cost of IL-17 biologics restricts their access by patients. Targeting IL-17A in mice using vaccines based on IL-17 displayed on Qß or CMV_TT_ has proven efficacious in preclinical models of myocarditis, [75], rheumatoid arthritis and multiple sclerosis [76] as well as psoriasis [77].

mAbs targeting TNF-α have demonstrated efficacy across a wide range of inflammatory diseases, such as psoriasis, Crohn’s disease, rheumatoid arthritis and ulcerative colitis [78, 79]. Unfortunately, patients treated with anti-TNF-α may develop resistance in the long term in addition to the large cost associated with the therapy. Hence, it may be attractive to generate a vaccine targeting TNF [80]. Two VLP-based vaccines have been described: one vaccine was based on HPV and displayed a TNF-derived peptide using biotin/streptavidin [80], and the other was based on full-length TNF or a TNF-derived peptide displayed on Qβ using SMPH chemistry and a free Cys introduced into TNF or the peptide [81]. Both strategies induced therapeutic antibodies capable of neutralizing TNF and abolishing disease in collagen-induced arthritis. Interestingly, the vaccine based on entire TNF linked to Qβ-VLPs induced antibodies that neutralized both transmembrane and soluble TNF. In contrast, the vaccine candidate based on 20 amino acids (a.a.) derived from the N-terminus of TNF (Qβ-C-TNF(4-23)) neutralized soluble TNF but not the membrane form. Nevertheless, both vaccines yielded specific antibodies that were capable of protecting mice from rheumatoid arthritis. In contrast, only mice immunized against full-length TNF (Qβ-C-TNF(1-156)) showed increased susceptibility to infection with Listeria monocytogenes as well as enhanced reactivation of latent *Mycobacterium tuberculosis*, while mice that received the peptide-based vaccine were not immunocompromised. Therefore, targeting soluble TNF-α alone can be an effective strategy for clinical translation that might overcome the risk of opportunistic infections [81]. The vaccine based on HPV and a TNF-derived peptide increased levels of anti-TNF antibodies by 1000-fold in comparison to the fusion peptide alone. As discussed above, this vaccine candidate also inhibited the development of collagen-induced arthritis in a mouse model [82]. Taking these findings into account, a clinical study was performed based on (Qβ-C-TNF(3-24)). The study showed disappointing results; however, the vaccine based on a murine TNF-derived peptide failed to induce TNF-neutralizing antibodies in humans (unpublished).

#### Allergy

Allergen-specific desensitization is currently the only available treatment for allergies. Such a strategy requires long-term application of allergens and can result in life-threatening anaphylactic shock [83]. Therapeutic nonreactogenic vaccines able to alleviate allergic symptoms after a few injections are therefore considered an attractive strategy. For the treatment of cat allergies, we have previously described a therapeutic vaccine that consists of the major cat allergen (Fel d 1) coupled to Qβ-VLPs. A single injection was capable of inducing protection against the type-1 allergic reaction. Additionally, allergen-induced systemic basophil degranulation was inhibited by allergen-specific IgG antibodies, a property that was enhanced by FcγRIIb [84]. Conjugating the allergen to VLPs not only increased allergen-specific IgG responses but also strongly reduced the ability of the allergens to cause allergic reactions, an important safety feature of this new specific immunotherapy approach [85]. Similar findings were made with allergens displayed on HBcAg [86].

To allow for rapid translation, we have developed an alternative strategy to treat Fel d1 allergy in humans by vaccinating cats against their own allergen. Using a conjugate vaccine based on CuMV_TT_, our results indicated tolerability and no overt toxicity in cats, and the vaccine also generated a strong and sustained specific IgG response with high affinity and neutralizing capacity. Humans and cats are expected to benefit from this treatment, which will reduce the risk of asthma in humans and will facilitate the interaction of owners with their cats [87].

Hirschberg et al. investigated the immune response induced following sensitization with the house dust mite *Dermatophagoides pteronyssinus* (Der p1) and vaccination with hybrid Ty-VLPs derived from the p1 protein of yeast retrotransposon carrying the immunodominant epitope Der p1. Their results demonstrate that the hybrid vaccine abrogated allergen-specific IL-5 production, and the effect was mediated by CD4^+^ T cells [88]. In an older study, it was shown that a Der p 1-derived peptide induced strong IgG responses in humans when it was chemically conjugated to Qβ-VLPs [89]. Recent evidence suggests that VLP-based vaccination against allergens may also be attractive for prophylactic intervention [90].

**Fig. 7:**
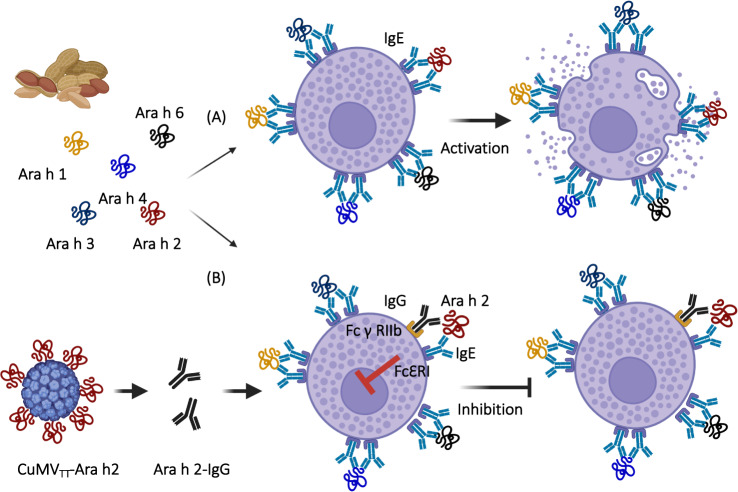
A cartoon illustrating the mechanism of inhibiting FcγRIIb on mast cells in a Peanut Allergy model. **A** Binding of peanut allergens to IgE bound to high-affinity FcƐ RI result in mast cells degranulation and allergic reactions. **B** Immunization with a VLP-based vaccine (CuMVTT-Arah2) induces specific-IgG antibodies for the single allergen Ara h2 forming an immune-complex that binds to FcγRIIb and inhibiting IgE-mediated signals. Created with BioRender.com

Peanut allergy is an increasingly frequent disease with a high burden, particularly in the US, and no treatment is currently available. CuMV_TT_-VLPs were used to display extracts of roasted peanut (Ara R) or purified single allergens Ara h1 or Ara h2 by chemical coupling and tested in a murine model of peanut allergy. One or two doses of all the generated vaccines could protect peanut-sensitized mice against anaphylactic reactions after challenge with the whole peanut extract. Surprisingly, all three vaccine candidates, also those based on single allergens, were able to confer protection against systemic as well as local challenge with the whole peanut extract, and all vaccines protected against eosinophil and mast cell infiltration in the gastrointestinal tract following an oral challenge with the complex extract [91]. These results demonstrate that vaccination against a single allergen can confer protection against challenge with a complex allergen mixture. Antibodies were established as the mode of action, as passive transfer of purified IgG or even a single monoclonal anti-Ara h 2 antibody conferred protection against allergic reactions in mice [91, 92]. The role of the Fc_γ_RIIb receptor on mast cells and basophils was also studied, and the obtained results demonstrate the critical role of this inhibitory receptor in blocking allergic reactions against the complex extract (Fig. 7). Briefly, the induction of high levels of specific IgG antibodies against a single allergen will result in the formation of immune complexes that will bind FcγRIIb, causing inhibition of IgE-mediated signals triggered also by other allergens [91, 93]. GMP material of the Ara h 2-based vaccine has been produced, and an IND in the US has been granted (Table 2) [94].

#### Pain

Nerve growth factor (NGF) is essential for early development of the nervous system, but at later stages, it becomes an essential mediator of pain. The mAb tanezumab has been shown to be efficacious in several human studies and is currently under registration [95], and a number of other mAbs are under development. Accordingly, developing a therapeutic vaccine targeting NGF may be of major interest. Indeed, we have shown the potent efficacy of a VLP-based vaccine against NGF to control pain in both rheumatoid arthritis and osteoarthritis in a preclinical study [96]. The developed vaccine was based on presenting murine NGF protein on CuMV_TT_ VLPs. However, further studies are essential to establish the safety of a vaccine targeting NGF.

#### Neurodegenerative diseases

##### Alzheimer’s disease

Alzheimer’s disease (AD) is a major contributor to dementia, which is a disease that increases rapidly in frequency with aging. According to the Alzheimer Association, AD affects approximately 6.2 million people over the age of 65 in America, and that number is expected to increase to 13.8 million by 2060 [97]. Additionally, the current cost of therapy, particularly nursing aid, reached > $250 billion in 2020 in the USA. Active immunization against amyloid plaques is a promising therapeutic strategy [98]. However, there are currently no approved prophylactic or therapeutic vaccines against AD.

After the original breakthrough studies that put a potential vaccine against AD in the spotlight [99], a first VLP vaccine candidate was developed based on Qβ and the N-terminal end of Aβ (CAD106) [100]. Only the first 6 a.a. were chosen since clinical studies with the original vaccine candidate Aβ1-42 formulated in adjuvants induced self-specific T cells that induced meningo-encephalitis in up to 10% of the patients [101]. Preclinical evaluation demonstrated that immunization with CAD106 in the absence of adjuvants caused efficient removal of plaques in amyloid precursor protein (APP) transgenic mice, reproducing the original findings obtained with Aβ1-42 formulated in adjuvants. In addition, no signs of Aβ-specific T cells were observed. Subsequent clinical studies in AD patients demonstrated removal of plaques without signs of meningoencephalitis or microhemorrhages [102, 103]. However, there is currently no definitive evidence for improved performance of CAD106-immunized AD patients.

Using our immunologically optimized VLPs (CuMV_TT_-VLPs), as well as the Aβ_1–6_ peptide, we established a vaccine against AD. As discussed earlier, CuMV_TT_ incorporates a TT epitope, which is proposed to enhance the response in aged individuals due to preexisting memory T_H_ cells against tetanus. The developed vaccine may be used in both prophylactic and therapeutic settings. CuMV_TT_-Aβ_1–6_ showed high immunogenicity in young and old mice, and prepriming against tetanus enhanced the response against Aβ1-42. Indeed, displaying Aβ_1–6_ on the surface of CuMV_TT-_induced antibodies exhibiting the right specificity as sera from immunized mice could recognize AD plaques on postmortem brain sections as efficiently as a monoclonal antibody raised against Aβ peptide 1–17 [77]. Recent efforts have utilized HBc to produce a personalized AD vaccine tailored to a specific patient. Researchers have constructed a platform by inserting SpyCatcher into the major immunodominant region (MIR) of truncated HBc which could assemble into uniform VLPs readily binding to different SpyTag epitopes such as Aβ(1-6), Aβ(1-15), cAβ(1-7) cEP1, and cEP2 from β-amyloid monomer or oligomers and T294, pTau396-404, and pTau422 from tau proteins. The results of one study indicated that HBc-S-pTau422 alleviated cognitive deficits as well as neuropathy progression in transgenic mice [104]. Strong immunogenicity and a good safety profile were also observed for alternative Qß- [105], HPV- [106] and retroviral particle- [107] based vaccine candidates.

Aβ may not be the only target for treating AD. Indeed, Maphis et al. reported the development of a therapeutic vaccine against tauopathies, including AD. Tau peptide, phosphorylated at threonine 181, was chemically linked to Qβ-VLPs (pT181- Qβ). The vaccine induced a robust and long-lived specific antibody response recognizing postmortem human brain sections and was capable of reducing soluble and insoluble pTau in both the cortex and hippocampus of transgenic mice [108]. Hence, vaccines against Aβ or Tau or perhaps a combination of both may be promising ways forward for the development of AD therapies or perhaps even prophylaxis.

##### Parkinson’s disease

Parkinson’s disease is considered the second most frequent neurodegenerative disease and is associated with movement disorders, cognitive impairment and progressive disability. The disease affects 1% of people older than 60 years [109]. Neutralization of toxic alpha-synuclein (a-syn) oligomers is proposed to play a role in stopping the spread of oligomers and aggregates in the brain as well as cellular toxicity. Doucet et al. proposed an active vaccination strategy based on conjugating short peptides targeting the C-terminal region of α-syn to the Qβ platform. The obtained results demonstrated the ability of the vaccine to induce high antibody titers in wild-type mice and a-syn transgenic mice and recognize Lewy bodies. Despite the ability of the induced antibodies to specifically recognize oligomeric α-syn in solution, the vaccine failed to confer significant changes in a transgenic mouse model, showing no effect on the behavioral phenotype compared to the control condition [110]. Whether the absence of preclinical efficacy was due to the particular mouse model used remains unclear, and such results indicate the need for optimized preclinical models, which may be a challenging task.

#### Cancer

Antibodies have been studied and used in several tumors and have been demonstrated to be essential players in antitumour immunity. The effector function of antitumour antibodies includes induction of apoptosis, interference with tumor signaling pathways, antibody-dependent cellular cytotoxicity (ADCC) or activation of complement. Even though most antitumour antibodies are passively administered in the form of mAbs, vaccination may be a promising alternative for the active induction of antitumour antibodies. Nevertheless, most vaccines against cancer aim to induce T cells rather than antibodies, and the vaccines described below could be more accurately described as “niche candidates”. Prominent examples of antibody-inducing cancer vaccines are based on AP205, which has been used preclinically as a platform for a therapeutic vaccine for the induction of antibodies for cancer therapy. Using the SpyTag/SpyCatcher technique, the authors decorated VLPs with human epidermal growth factor receptor-2 (HER-2), a classical target for mAb therapy. Using this strategy, they could overcome B-cell tolerance, and potent anti-HER2 IgG antibodies were induced, hindering the progression of breast carcinoma tumors expressing HER-2 in mice [111]. Furthermore, overexpression of the xCT protein in triple-negative breast cancer tumors has been used as a target epitope and inserted into MS2-VLPs. The vaccine elicited a potent specific anti-IgG2a response, hampered tumor growth and prevented metastasis in an established 4T1 mouse model [91].

**Fig. 8:**
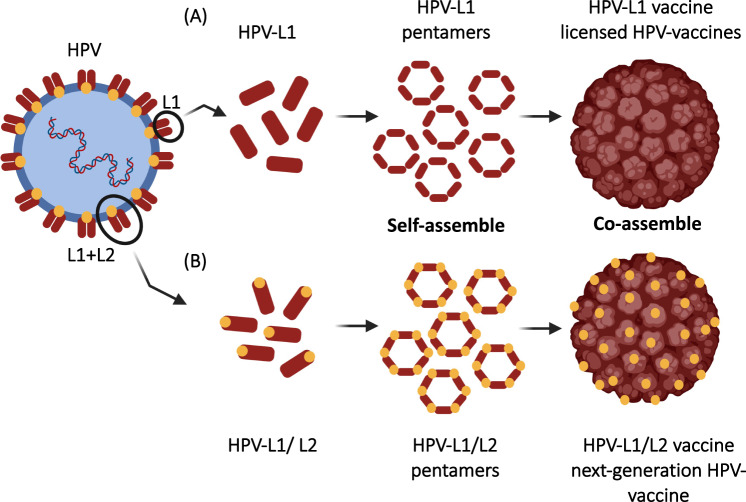
L1 and L2-capdid proteins of HPV **A** L1-capsid protein of HPV is a highly immunogenic epitope that can assemble into VLPs, example (licensed prophylactic HPV-based vaccines: Cervarix®, Gardasil® and Gardasil9®). **B** A chimeric HPV vaccine expressing both capsid proteins L1 and L2. Created with BioRender.Com

Human papillomavirus-related cancers account for ~4.5% of all types of malignancies and have been shown to affect more than half a million people every year [112]. Cancers associated with HPV include cervical carcinoma (99.7%) and squamous cell carcinoma of the vagina, anus, penis, vulva and oropharynx [113, 114]. The capsid L1 protein of HPV is a highly immunogenic epitope targeted in licensed prophylactic HPV vaccines (discussed later in 4). The structural L1 protein can self-assemble into VLPs that are morphologically similar to the parental native virus (Fig. 8). The minor capsid protein L2 is a highly conserved protein among HPV types and accordingly can be considered an appropriate target for the development of a next-generation Pan-HPV vaccine. However, L2-based vaccines have been shown to induce lower levels of neutralizing antibodies than L1-based vaccines [115]. Schellenbacher et al. have constructed a chimeric HPV vaccine expressing both L1 and L2 proteins (HPV16 L1-HPV16 L2 (chimera 17–36)) and adjuvanted VLPs induced broad-spectrum neutralizing antibodies directed against HPV types that are divergent from HPV16. These findings suggest the possibility for such a chimeric vaccine to protect against mucosal infection caused by high-risk, low-risk and beta HPV-associated diseases [116]. Clinical studies on RG1-VLP as another attempt to use L2 as a vaccine candidate will soon begin. RG1-VLPs contain the conserved amino acids 17–36 of HPV16L2, which are fused closely in the immunodominant surface loop of HPV16L1-VLP. Immunization experiments in small animal models have confirmed cross-protection against medically relevant high- and low-risk HPV types [117]. AAVLP-HPV is another chimeric vaccine that was constructed to display the L2 epitope [10–13, 15–25, 32, 118–121] from HPV16 and 31 onadeno-associated virus-derived VLPs. Immunization of mice and rabbits with these VLPs formulated in Montanide adjuvant induced specific antibody titers that were able to neutralize infection with several HPV types in a pseudovirion infection assay[122]. This chimeric vaccine successfully completed a phase I clinical trial (Table 2).

### VLP-based therapeutic vaccines for the induction of protective T cells in a therapeutic regimen

VLPs decorated with T-cell epitopes are efficient tools for eliciting T_H_1 as well as cytotoxic T-cell lymphocytes (CTLs), even though VLPs do not incorporate genetic material [123]. Exogenous antigens preferably enter the major histocompatibility class-II (MHC-II) pathway to prime CD4^+^ T cells. However, previous studies have shown that particulate VLPs can be successfully taken up by APCs and cross-presented to enter the MHC-I pathway as well [124, 125]. Loading MHC-I can be achieved via a transporter associated with the antigen processing (TAP)-independent endosomal pathway or TAP-dependent endosome-to-cytosol pathway. Combining VLPs with stimuli for APCs results in CTL and T_H_1 responses [126, 127]. From all TLR ligands tested in mice, stimulation of TLR3, TLR7/8 and in particular TLR9 was most effective, while other TLRs, such as TLR2 and TLR4, had little ability to stimulate the CTL response [128]. Furthermore, Qß-VLPs loaded with TLR ligands, such as RNA or CpGs, were efficient at inducing both CTL and T_H_1 responses but failed to do so when loaded with polyglutamate or delivered empty [129]. Unexpectedly, we have shown that VLPs and TLR ligands can be delivered separately without a need for physical linkage for the generation of CTL responses in vivo as long as TLR ligands are formulated as particles [130, 131]. Depot-forming adjuvants may provide further opportunities to enhance the induced T-cell response [132].

#### Cancer

##### Cancer caused by human papilloma virus (HPV)

Prophylactic HPV vaccines aim to generate a humoral response against the late proteins L1 and L2. Accordingly, such vaccines would not show efficacy in a therapeutic setting, as virion capsid proteins are not detected in virus-infected proliferating cells. The main aim of an HPV therapeutic vaccine is to eliminate precancerous lesions and persistent HPV infection [133]. The early E-proteins (E6 and E7) are transcription factors that are responsible for driving the proliferation of infected cells [134]. Both proteins are consequently expressed at high levels in tumor cells, which makes them ideal targets for therapeutic vaccines. An increased CD4^+^:CD8^+^ ratio in the stroma, CD4^+^ T-cell response to E2 protein and E6- and E7-specific CD8^+^ T-cell infiltration have been detected in spontaneous regression of cervical interepithelial neoplasia (CIN) [133]. Several different preclinical approaches have been followed to develop a therapeutic HPV vaccine, including the use of VLPs. Some studies designed a T-cell-based vaccine by targeting the oncogenic proteins E6 and E7, and others developed a chimeric vaccine incorporating both capsid and oncogenic proteins. For example, Greenstone et al. demonstrated the ability of HPV-VLPs to induce cell-mediated immunity by generating chimeric VLPs consisting of the major capsid protein L1 plus the entire nonstructural E7 or E2 fused to minor capsid protein L2 [135]. HPV16 L1/L2-HPV16 E7 chimeric VLPs protected wild-type and MHC-II-deficient mice (lacking Th cells) from TC-1 tumor challenge [135]. Another study utilized a modified rabbit hemorrhagic disease virus VLP (RHDV-VLP) as a vaccine platform decorated with the E7_48–57_ peptide. The developed vaccine was tested by using the TC-1-cell line expressing both E6 and E7 antigens in a murine model. The authors combined the vaccine with an anti-CTLA-4 checkpoint inhibitor or with anti-CD25 for Treg depletion. The results indicated a 50% reduction in tumor burden and a significantly enhanced survival rate [136]. We have shown that Qß-VLPs displaying or mixed with E7 protein could induce protection against tumor growth and enhance survival for more than 80% of vaccinated mice [131]. Another study generated a VLP-E7 vaccine incorporating a long E7 protein fragment into bursal disease virus VLPs. The VLP-E7 vaccine was tested in humanized transgenic mice expressing human HLA-A2 inoculated with the TC1/A2 cancer cell line in a therapeutic setting. The results showed complete eradication of established tumors as well as long-lasting immune responses [137].

##### Melanoma

A number of preclinical experiments have explored the efficacy of using VLPs as a therapeutic vaccine against melanoma.

Qβ-VLPs loaded with type-A CpGs (Qβ(G10), also called CMP-001) do not contain any tumor antigen and have been tested for anti-melanoma response upon intratumour injection. Sabree et al. demonstrated that CMP-001 induces the formation of anti-Qβ antibodies that opsonize Qβ VLPs, which are subsequently taken up by plasmacytoid dendritic cells (pDCs), leading to cytokine secretion and an antitumour T-cell response [138]. CMP-001 has been used in clinical trials, as shown in Table 2. In a recently published paper, Melhim et al. showed that CMP-001 alone or in combination with pembrolizumab (PD-1 inhibitor) in patients with advanced melanoma is tolerable and capable of reversing anti-PD1 resistance therapy with durable and strong clinical responses [139].

The Qβ(G10)MelanA vaccine was developed using a chemical coupling method, and potential efficacy was demonstrated in a phase I/II study in stage II-IV melanoma patients. More than 60% of the treated patients generated specific effector and memory T-cell responses as well as high IFN-γ, TNF-α and IL-2 cytokine production [140]. Recently, we have shifted our efforts toward developing personalized VLP-based vaccines targeting the patient’s tumor-specific epitopes for effective T-cell responses. Given that targeting a single antigen allows tumor cells to relapse by downregulating this single antigen, we designed a multitarget vaccine in a challenging transplanted murine melanoma model. Our results demonstrate that targeting both germline and mutated epitopes enhances the induced antitumour response [141]. We also tested the depot effect of microcrystalline (MCT) adjuvant in combination with CuMV_TT_-p33 vaccine. The results showed that MCT polarizes the response toward TH1 and enhances the induced antitumour response [132].

##### Mammary carcinoma

Since we have shown enhanced efficacy using the multitarget vaccine strategy in melanoma, we have expanded these findings to an aggressive metastatic breast carcinoma murine model. To further improve the active immunotherapy, we designed a personalized multitarget vaccine displaying elongated neoantigens using Qβ-VLPs packaging a TLR9 ligand by integrating mass spectrometry-based immunopeptidomics and whole-exome sequencing. Vaccination with long neoantigens was more effective than vaccination with short neoantigens, and the antitumor effect could significantly repolarize the tumor microenvironment, reduce lung metastasis and enhance survival [142].

#### Acquired Immunodeficiency Syndrome (AIDS)

Since its discovery as the etiological agent underlying acquired immunodeficiency syndrome (AIDS) in the 1980s, HIV has become a major global public health concern. Despite the ability of antiviral agents to keep the virus at bay, there is an urgent need for a therapeutic vaccine against HIV to eliminate long-term persistant HIV in patients on antiretroviral therapy. Insights into the pathogenesis of HIV-1 suggest that T-cell immunity plays a crucial role in controlling the acute phase of HIV infection [143], and long-term studies indicated that CD8^+^ CTL responses are associated with the control of HIV replication and infection clearance [144].

In 1996, Wolf et al. constructed and expressed a chimeric HIV-1 VLP vaccine. The 3^rd^ variable region (V3) or CD4-binding domain of gp120 was inserted into Pr55^gag^-VLPs; this insertion did not interfere with the assembly ability of the VLPs. Mice were immunized using different routes and different chimeric Pr55^gag^/V3 VLPs without any adjuvants. The results showed a strong MHC-I (D^d^) CTL response against a known epitope within the V3 region [145]. Furthermore, it has been shown that Gag-VLP vaccines were capable of inducing interferon alpha (IFN-α) in treated monocyte-derived dendritic cells (DCs) with increased expression of the mRNA encoding the proteins APOBEC3G and APOBEC3F, known inhibitors of HIV-1 [146]. In addition, yeast-derived VLPs containing the HIV V3 loop have been shown to be processed by murine dendritic cells for presentation in association with MHC class I [147].

## Clinical development

Several prophylactic and therapeutic VLP-based vaccines are currently in the clinical development stage. We list the clinical trials related to the above discussed diseases in Table 2.

**Table 2:** List of VLP-based vaccines in clinical trials.

Disease	Total no. of ongoing clinical trials	General Information		
COVID-19	7	NCT No.	Phase	VLP-based vaccine
		NCT04962893	2	SARS-CoV-2 VLP vaccine
		NCT04773665	1	VBI-2902a
		NCT04818281	1	SARS-CoV-2 VLP vaccine
		NCT04839146	1	ABNCoV2 vaccine
		NCT05040789	3	CoVLP formulation
		NCT05137444	2/3	LYB001 vaccine
		NCT05125926	1	LYB001 vaccine
Influenza	22	NCT No.	Phase	VLP-based vaccine
		NCT00903552	2	Influenza A vaccine
		NCT01561768	2	Novavax quadrivalent vaccine
		NCT01014806	2	Influenza VLP vaccine
		NCT01072799	2	A/H1N1 2009 influenza VLP vaccine
		NCT01596725	1	Monovalent avian influenza VLP (H5N1) vaccine, with/without adjuvant
		NCT01594320	1	Monovalent avian influenza VLP (H5N1) vaccine, with/without adjuvant
		NCT00754455	2	Influenza VLP vaccine (recombinant)
		NCT03321968	3	Quadrivalent VLP influenza vaccine
		NCT03301051	3	Quadrivalent VLP influenza vaccine
		NCT02307851	2	Quadrivalent VLP influenza vaccine
		NCT02022163	1	H7 VLP vaccine + Alhydrogel
		NCT02233816	2	Quadrivalent VLP vaccine
		NCT00519389	1/2	H5N1 VLP vaccine
		NCT01897701	1	Monovalent avian influenza VLP (H7N9) vaccine
		NCT02078674	1/2	Monovalent avian influenza VLP (H7N9) vaccine
		NCT02768805	2	Quadrivalent VLP vaccine
		NCT01991561	2	H5 VLP vaccine with adjuvant
		NCT01991587	1/2	Quadrivalent VLP influenza vaccine
		NCT02236052	2	Quadrivalent VLP vaccine
		NCT00984945	1	H5 VLP pandemic influenza vaccine
		NCT01657929	1	H5-VLP vaccine with/without adjuvant
		NCT03739112	3	Quadrivalent VLP vaccine
Malaria	21	NCT No.	Phase	VLP-based vaccine
		NCT05357560	1	Matrix-M with RH5.2 VLP and/or R21
		NCT05252845	2	R21/Matrix-M vaccine
		NCT00587249	1	Malaria ICC-1132
		NCT04327440	–	RTS,S/AS01
		NCT02992119	2	RTS,S/AS01
		NCT04319380	3	RTS,S/AS01
		NCT03143218	3	RTS,S/AS01
		NCT00197067	1	RTS,S/AS02D and RTS,S/AS02A
		NCT01556945	1/2	RTS,S/AS02
		NCT00197054	2	RTS,S/AS01B, RTS,S/AS02A
		NCT00197041	2	RTS,S/AS02A
		NCT01883609	1/2	RTS,S/AS01B
		NCT02252640	1/2	RTS,S/AS01B
		NCT03824236	2	RTS,S/AS01E (SB257049)
		NCT00075049	1/2	RTS,S/AS02A, RTS,S/AS01B
		NCT04661579	2	RTS,S/AS01E
		NCT00197028	2	RTS,S/AS02D
		NCT03162614	2	RTS,S/AS01E, RTS,S/AS01B
		NCT03276962	2	RTS,S/AS01E
		NCT00307021	2	RTS,S
		NCT00360230	2	different formulation of RTS,S
Alzheimer	6	NCT No.	Phase	VLP-based vaccine
		NCT00956410	2	CAD106
		NCT01023685	2	CAD106
		NCT00795418	2	CAD106
		NCT00411580	1	CAD106
		NCT01097096	2	CAD106
		NCT00733863	2	CAD106
AIDS	1	NCT No.	Phase	VLP-based vaccine
		NCT00001053	1	HIV p17/p24:Ty-VLP
Melanoma	9	NCT No.	Phase	VLP-based vaccine
		NCT03084640	1	CMP-001
		NCT02680184	1	CMP-001
		NCT03618641	2	CMP-001
		NCT04698187	2	CMP-001
		NCT04695977	2/3	CMP-001
		NCT04401995	2	CMP-001
		NCT04708418	2	CMP-001
		NCT04387071	1/2	CMP-001
		NCT02554812	2	CMP-001
HPV	49	NCT No.	Phase	VLP-based vaccine
		NCT00365716	2	Quadrivalent HPV (Types 6,11,16,18) L1 VLP vaccine
		NCT01984697	3	V503 (9-valent [HPV] L1 [VLP] vaccine)
		NCT00635830	1	Quadrivalent HPV (Types 6, 11, 16, 18) recombinant vaccine
		NCT01254643	3	9-Valent HPV L1 (VLP) vaccine
		NCT02740777	2	HPV-16/18 vaccine
		NCT05334706	–	9vHPV/Gardasil-9™
		NCT00834106	3	Quadrivalent HPV (Types 6, 11, 16, 18) recombinant vaccine
		NCT00851643	1	Octavalent HPV vaccine
		NCT04199689	3	9vHPV vaccine
		NCT01101750	4	Quadrivalent HPV (Types 6, 11, 16 and 18) vaccine
		NCT00365378	2	HPV 16 L1 vaccine
		NCT00543543	3	V503 vaccine
		NCT03903562	3	V503 vaccine
		NCT01651949	3	9vHPV vaccine
		NCT00496626	3	Quadrivalent HPV (Types 6, 11, 16, 18) recombinant vaccine (Gardasil®)
		NCT00260039	2	Octavalent HPV vaccine
		NCT04711265	–	Quadrivalent HPV (Type 6, 11, 16 and 18) L1 VLP vaccine
		NCT00339040	2	Quadrivalent HPV (Types 6, 11, 16, 18) L1 (VLP) or Quadrivalent HPV vaccine (QHPV)
		NCT02733068	3	HPV-16/18 vaccine
		NCT01073293	3	V503 vaccine
		NCT00988884	3	V503 vaccine
		NCT03158220	3	V503 vaccine
		NCT00520598	2	V505 vaccine
		NCT02653118	–	V503 vaccine
		NCT02114385	3	V503 vaccine
		NCT01047345	3	V503 vaccine
		NCT03929172	1	AAVLP-HPV vaccine
		NCT00092547	3	V501 vaccine
		NCT02576054	3	V501 vaccine
		NCT05031078	4	Gardasil
		NCT00693966	2	MEDI-517 HPV-16/18 VLP vaccine
		NCT00693615	2	MEDI-517 HPV-16/18 VLP vaccine
		NCT03296397	–	Quadrivalent HPV vaccine
		NCT00092495	3	V501, Gardasil, HPV (Types 6, 11, 16, 18) recombinant vaccine
		NCT00092482	3	V501, Gardasil, HPV (Types 6, 11, 16, 18) recombinant vaccine
		NCT00092534	3	Gardasil, HPV (types 6, 11, 16, 18) recombinant vaccine
		NCT05285826	3	9vHPV vaccine
		NCT01544478	4	V501 vaccine
		NCT00411749	2	Quadrivalent HPV (types 6, 11, 16, 18) recombinant vaccine (V501)
		NCT05045755	-	Recombinant HPV Bivalent (types 16, 18) vaccine (*Escherichia coli*)
		NCT02710851	2	HPV vaccine
		NCT00128661	3	HPV 16/18 L1 VLP/AS04 vaccine
		NCT01735006	3	HPV vaccine HEV vaccine
		NCT00586339	2	Cervarix
		NCT03546842	3	9vHPV vaccine
		NCT00337428	3	Quadrivalent HPV (types 6, 11, 16, 18) recombinant (qHPV) vaccine
		NCT00337428	3	Quadrivalent HPV (types 6, 11, 16, 18) recombinant (qHPV) vaccine
		NCT04635423	3	V503 vaccine
		NCT04508309	3	Cecolin® Gardasil®

All registered clinical trials are listed as available on the www.clinicaltrial.gov website.

## Marketed VLP-based vaccines

### Human papilloma virus vaccine

Gardasil® was the 1^st^ vaccine to be approved as a prophylactic vaccine against human-papilloma virus (HPV). Gardasil9®, covering 9 rather than 4 serotypes, has replaced Gardasil® in the US. The Gardasil®, Gardasil9® and Cervarix® vaccines are recombinantly manufactured VLPs of the L1-HPV epitope (Table 3). However, Gardasil9® contains a larger total antigenic load than Gardasil® to compensate for the larger number of different co-formulated VLPs [148]. Gardasil protects against 4 types of HPV (6, 11, 16 and 18), and Gardasil9® protects against 5 additional types (31, 33, 45, 52 and 58) [149]. The vaccines have been designed to elicit virus-neutralizing antibodies with the goal of preventing the initial HPV infection. These marketed vaccines have been shown to provide 100% protection against cervical precancerous as well as genital wart development with a protective effect expected to last a minimum of 4.5 years [150]. HPV vaccines are safe, and local adverse reactions such as swelling, pain and redness are mostly mild and reversible in the short term. Systemic reactions are rare and may involve nausea, fever, headache and fatigue [151]. The current cost of licensed HPV vaccines in yeasts or insect expression systems may be considered a barrier to their substantial implementation worldwide. Recent efforts to express low-cost HPV vaccines in *E*. *coli* have been successful. Cecolin® is a bivalent L1-HPV vaccine against HPV16 and HPV18 that was successfully expressed in *E*. *coli* and has shown high immunogenicity in mice, goats and rabbits [152]. On December 30, 2019, Cecolin® was officially approved by The National Medical Products Administration for use in China and was launched there in May 2020. In October 2021, the WHO granted prequalification for Cecolin®, making it the 1^st^ China-manufactured HPV vaccine that received WHO prequalification [153].

**Table 3:** List of the currently approved HPV prophylactic vaccines based on VLPs on the market

Trade name	Targeted epitope	Expression system	Adjuvant	Manufacturer
Cervarix® (bivalent vaccine)	L1 HPV 16 L1 HPV 18	Baculovirus Hi-5 baculovirus	ASO4:AI(OH)_3_ MPL	GSK
Gardasil® (quadrivalent vaccine)	L1 HPV 6 L1 HPV 11 L1 HPV 16 L1 HPV 18	Yeast (*S. cerevisiae*)	Amorphous, Aluminum, hydroxyphosphate sulfate	Merck Sharp & Dohme Corp
Gardasil9® (nonavalent vaccine)	L1 HPV 6 L1 HPV 11 L1 HPV 16 L1 HPV 18 L1 HPV 31 L1 HPV 33 L1 HPV 45 L1 HPV 52 L1 HPV 58	Yeast (*S. cerevisiae*)	Amorphous, Aluminum, hydroxyphosphate sulfate	Merck Inc.
Cecolin®	L1 HPV 16 L1 HPV 18	*E*. *coli*	–	INNOVAX

### Hepatitis B virus (HBV) vaccine

Several HBV vaccines against the potentially life-threatening disease hepatitis B are commercially available. HBV infection can cause a chronic infection and danger people with cirrhosis and liver cancer. The available vaccines show 98–100% protection against HBV.

The first-generation HBV vaccine was approved in 1981 based on the work of Bulmberg, who was awarded the Nobel Prize for the discovery of the Australian Antigen, now called hepatitis B surface antigen (HBsAg), in the serum of an infected patient [154]. The unique vaccine was based on obtaining HBsAg directly from human carriers. However, due to biosafety concerns, this blood-derived vaccine was replaced with an improved recombinant version of HBsAg in 1986 using the recombinant DNA method and production in yeast cells and currently also in mammalian cells. The HBV recombinant vaccine (second-generation) was initially produced using the yeast system. Available yeast-derived HBV vaccines are based on the self-assembly of HBsAg monomers into VLPs. Several studies have shown that purified yeast-derived HBsAg appears as ∼22 nm VLPs [155–157]. Sixty to seventy percent of the formed HBsAg VLPs consist of HBsAg monomer proteins, while the rest consist of lipids [158]. Overall, the formed VLPs are highly immunogenic and capable of eliciting potent neutralizing antibodies in addition to being a safe vaccine lacking any viral genome [159, 160]. Vaccination with a single antigen (small S antigen of HBsAg) has some limitations, including the prolonged time required to achieve seroprotection. Thirty to forty percent of adults reached seroprotection following 2 doses of the vaccine, but 10% may fail to achieve this even after administration of a 3^rd^ dose [161]. The recently FDA-approved third-generation HBV vaccine PreHevBrio expresses three surface antigens of HBV, S, pre-S1 and pre-S2, and has been manufactured in mammalian cells. Phase III clinical trials confirmed high immunogenicity of the vaccine even in older people and those with chronic conditions [162]. The study concluded noninferiority of the vaccine in seroprotection rate when compared to a single antigen vaccine 4 weeks following the 3^rd^ dose. The seroprotection rate was higher after the administration of 2 or 3 doses than after vaccination with the classical HBV vaccine (the control group). Rapid induction of a protective antibody profile was reported in more than 90% of participants after 2 doses; moreover, a good safety profile was observed. Table 4 summarizes the different HBV vaccines available, and Table 5 outlines the main differences between the second- and third-generation HBV vaccines.

**Table 4:** Some FDA-approved vaccines for HBV based on VLPs on the market

Trade name	Targeted epitope	Expression system	Adjuvant	Manufacturer
Engerix-B® Dec 2018	S antigen	Yeast (*P*. *pastoris*)	Aluminum hydroxide	GSK
Recombivax HB® Dec 2018	S antigen	Yeast (*S. cerevisiae*)	Aluminum sulfate	Merck Sharp & Dohme Corp
Heplisav-B® Nov 2017	S antigen	Yeast (*H. polymorpha*)	1018 CpGs	Dynavax Technologies Corp
PreHevbrio® (Sci-B-Vax) Nov 2021	S antigen, pre-S1 antigen, pre-S2 antigen	Mammalian (CHO cells)	Aluminum hydroxide	VBI Vaccines

**Table 5:** Comparison between second- and third-generation HBV vaccines

Comparison	Second-generation HBV vaccine	Third-generation HBV vaccine
Viral antigens	S-antigen	S-antigen Pre-S2 antigen Pre-S1 antigen
Adjuvant	Aluminum hydroxide or CpGs 1018	Aluminum hydroxide
Dose	10, 20 or 40 μg	10 μg
Trademarks	Engerix-B®, Recombivax HB® and Heplisav-B®	PreHevbrio® (Sci-B-Vax)

### Hepatitis E virus (HEV) vaccine

Hepatitis E virus is responsible for more than 50% of acute viral hepatitis in countries such as India China, Kenya, Sudan, Nepal and Bangladesh [163]. Studies have estimated that ∼35 million cases occur annually worldwide, with an average mortality rate of 0.2–4% and up to 25% in pregnant women [164]. HEV is a nonenveloped virus with a size of 27–35 nm and is divided into four different genotypes, I, II, III and IV [165]. Genotypes I and II are restricted to humans, while genotypes III and IV infect other mammals who can subsequently transmit the virus to humans. Despite this genetic diversity, all HEVs belong to the same serotype. The virus’s positive-stranded RNA genome contains three open reading frames, one of which encodes the viral capsid protein, the main target of neutralizing antibodies [166].

HEV239 (Hecolin®) was licensed as a vaccine against HEV in China in 2012. The vaccine is a recombinant VLP-based vaccine developed by Xiamen Innovax Biotech Co., Ltd. (China), and the encoding gene is from the ORF2 HEV genotype I strain. The produced recombinant protein comprising amino acids 368–606 (p239) 4-5 produced in an *E*. *coli* expression system, refolded and self-assembled into VLPs 20–30 nm in size [167]. The formed VLPs are highly immunogenic in both mice and rhesus macaques. Hecolin® has shown a good safety profile and 100% efficacy in humans. Studies are currently underway to assess vaccine safety and efficacy in high-risk groups for potential global use, as recommended by the WHO [168].

### Malaria vaccine

RTS,S/AS01 (Mosquirix™) is considered the most advanced vaccine targeting the preerythrocytic malaria stage to date. The monovalent recombinant vaccine targets a fragment of circumsporozoite protein (CSP) protein, the major component of *P. falciparum* coat protein. The CSP protein fragment was fused to HBsAg as a carrier. In RTS,S, R: stands for the 19 central tandem repeats, T: stands for T epitope at the C-terminus, first S stands for modified surface antigen and the second S refers to the nonmodified surface antigen that allows for the presentation of CSP on the particle’s surface in mosaic HBsAg particles [169]. The construct assembles into VLPs and is formulated with AS01, a potent adjuvant. RTS,S/AS01 has been evaluated in a phase III clinical study; however, the results revealed only partial protection with some concerning safety issues, in particular a possible increase in mortality rates in young females [170]. RTS,S mosaic particles contain a 4-fold molar excess of nonmodified HBsAg subunits compared to CSP-HBsAg fusion protein. In contrast, the next-generation RTS,S/AS01 vaccine (R21) contains only CSP-HBsAg fusion protein and R21 therefore displays more CSP epitope on the surface and is capable of inducing sterile protection in mice [171]. Several late-stage clinical trials are ongoing to test the safety and efficacy of the next-generation R21 as well as to improve the efficacy of the first-generation RTS,S (Table 2). On October 6, 2021, the WHO recommended the widespread use of the RTS,S/AS01 vaccine among children in sub-Saharan Africa as well as in regions with moderate to high *P. falciparum* transmission [172].

## Concluding remarks

VLPs exhibit a unique combination of high immunogenicity combined with excellent safety profiles, rendering them ideal platforms for vaccine design. Since the first description of VLP-based HBV vaccines, the field has made notable progress, and VLPs are now the basis of many marketed vaccines, such as those against HPV and HEV. Very exciting novel developments have been made regarding the use of VLPs as display platforms, not only for pathogen-derived antigens such as CSP displayed on HBsAg for immunization against malaria but also against self-molecules, such as Aβ for vaccines against Alzheimer’s disease or cytokines for the treatment of chronic diseases in companion animals and humans. We therefore expect several new products, both for humans and animals, to reach the market in the next few years.
